# Unmasking the Intricate Association Between Takotsubo Syndrome, Atrial Fibrillation, and Diabetes Mellitus: A Case Report and Literature Review

**DOI:** 10.7759/cureus.66516

**Published:** 2024-08-09

**Authors:** Adam Bouzhir, Reda Taoufik Benchekroun, Nawal Doghmi, Jamila Zarzur, Mohamed Cherti

**Affiliations:** 1 Cardiology B Department, University Hospital Center Ibn Sina, Mohammed V University of Rabat, Rabat, MAR

**Keywords:** tako-tsubo syndrome, non obstructive coronary artery disease, left ventricular dysfunction, catecholamines, stress, atrial fibrillation, diabetes mellitus

## Abstract

Takotsubo syndrome (TTS) is characterized by transient left ventricular dysfunction without obstructive coronary artery disease, often mimicking acute coronary syndrome. Its association with diabetes mellitus and arrhythmias, such as atrial fibrillation (AF), suggests potential shared pathophysiological mechanisms. We report the case of a 76-year-old woman with diabetes who developed sudden, severe chest pain and palpitations after cataract surgery. Initial EKG showed ST-segment elevation, and laboratory tests revealed elevated high-sensitivity troponin, inflammatory markers, and diabetic ketoacidosis (DKA). Despite acute coronary syndrome symptoms, coronary angiography showed no significant obstruction. Transthoracic echocardiography revealed left ventricular apical akinesia and a moderately reduced ejection fraction. A cardiac MRI a month later demonstrated complete recovery of left ventricular function and spontaneous resolution of AF tachycardia. This case highlights a rare presentation of TTS in a diabetic patient with AF and DKA. The spontaneous resolution of AF and recovery of left ventricular function underscore the complex interplay between these conditions. Further research is needed to explore the mechanisms linking TTS with diabetes and AF to improve clinical management and outcomes.

## Introduction

Takotsubo syndrome (TTS), also known as stress-induced cardiomyopathy or “broken heart syndrome,” is marked by transient left ventricular dysfunction without obstructive coronary artery disease [1,2]. While research often explores TTS’s etiology, focusing on catecholamine excess, microvascular dysfunction, and myocardial stunning [3,4], it typically presents with symptoms resembling acute coronary syndrome, such as chest pain, dyspnea, and electrocardiographic changes [5,6]. Although TTS’s physiopathology is well documented, its interaction with conditions like diabetes mellitus and arrhythmias suggests potential common pathophysiological mechanisms or cause-effect relationships [7,8-14]. We present a case of atypical TTS with persistent atrial fibrillation (AF) tachycardia and diabetic ketoacidosis (DKA) in a diabetic female patient, highlighting the complex association between these conditions and underscoring the need to include TTS in the differential diagnosis for acute coronary syndrome.

## Case presentation

A 76-year-old female with a history of diabetes mellitus presented with sudden-onset severe chest pain at rest, which began five to six hours postoperatively after cataract surgery. This was accompanied by palpitations, which were initially neglected by the patient. However, due to the persistence of symptoms, the patient sought emergency care 30 hours after the onset of pain. Additionally, she reported a dry cough lasting about 10 days. Clinical examination revealed a conscious patient with slight chest pain and a rapid, irregular cardiac rhythm of 155 beats per minute, without clinical signs of heart failure.

**Figure 1:**
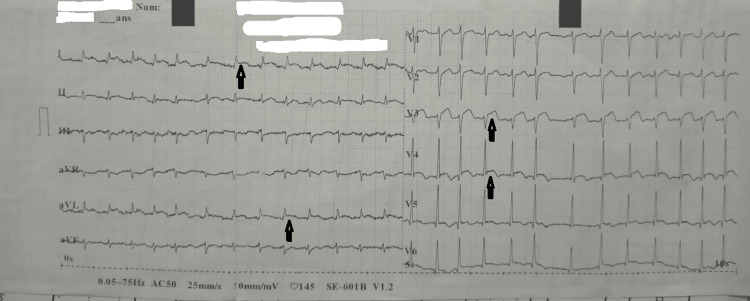
EKG at admission to the emergency department The EKG shows AF with slight ST-segment elevation in the apico-lateral leads (black arrow) and biphasic T waves in the low lateral leads.

The EKG performed in the emergency department revealed AF tachycardia with ST-segment elevation in the apico-lateral leads (Figure 1).

**Figure 2:**
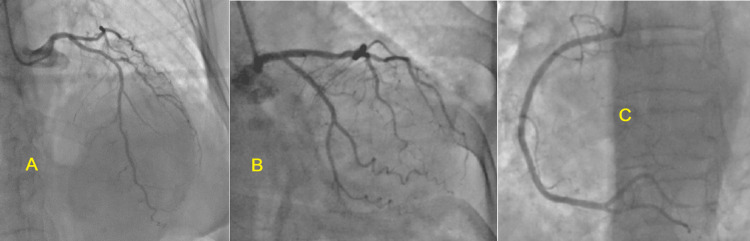
Coronary angiography images showing the left (A and B) and right (C) epicardial coronary networks, which are free from significant lesions

Thoracic radiography showed slight cardiomegaly and a slight reticulonodular interstitial syndrome. Laboratory investigations at admission revealed a markedly elevated high-sensitivity cardiac troponin (hs-troponin) level of 3,859 ng/ml, a positive CRP of 246 mg/l, and hyperleukocytosis of 18,600 cells/mm³ with neutrophil predominance. Given the suspicion of acute coronary syndrome, the patient underwent emergent coronary angiography, which surprisingly revealed no significant coronary artery stenosis or obstructive lesions (Figure 2A, 2B, 2C).

**Figure 3:**
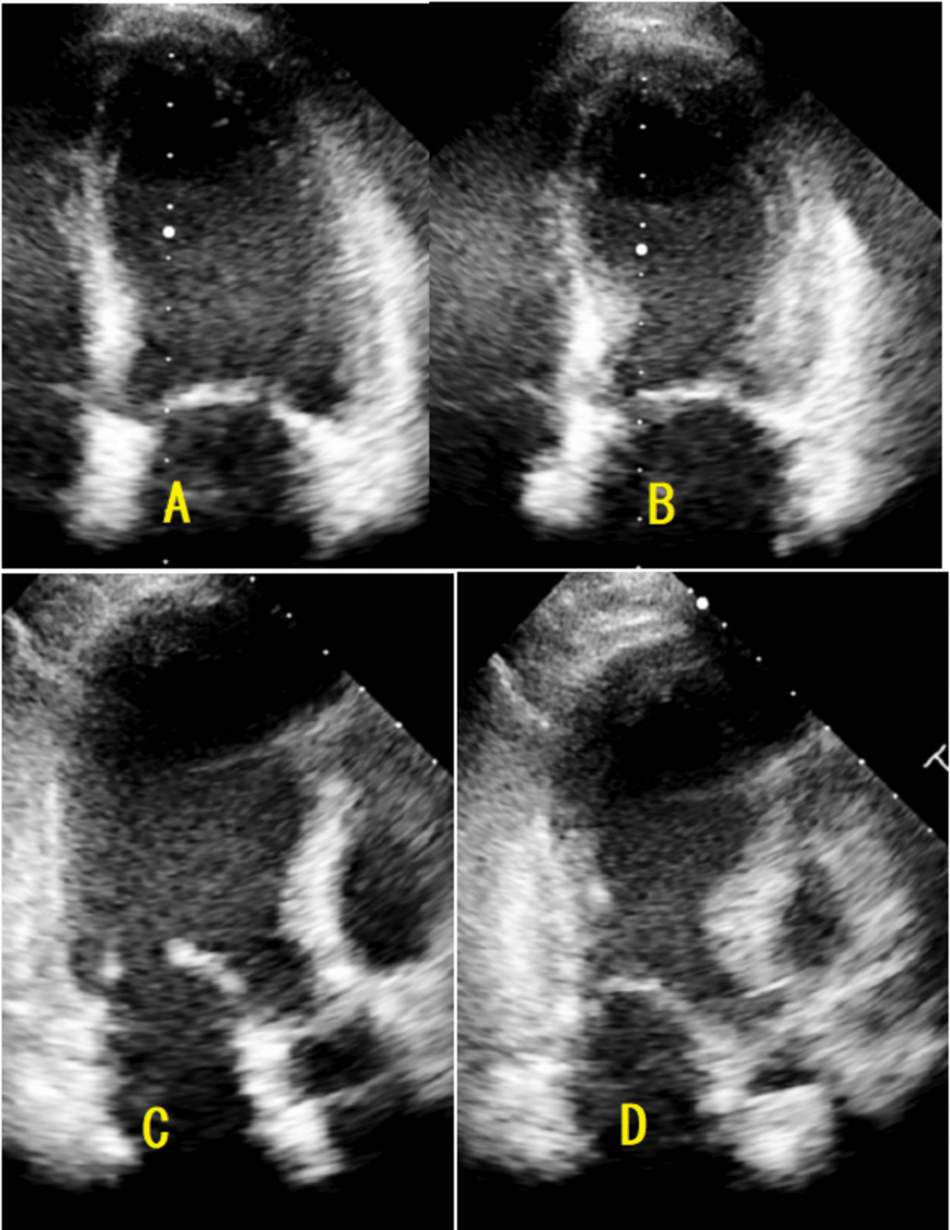
Echocardiographic images showing apical four-chamber and three-chamber views of the patient, highlighting classic apical ballooning with akinesia and hyperdynamic basal segments Apical four-chamber view in diastole (A) and systole (B) and three-chamber view in diastole (C) and systole (D).

Transthoracic echocardiography revealed a left ventricle with normal dimensions, akinesia of the apex and adjacent segments extending partially into the middle segments of various walls, and a moderately reduced ejection fraction of 43% (Figure 3A, 3B, 3C, 3D).

The patient was treated with oral anticoagulants, beta-blockers, and angiotensin-converting enzyme inhibitors, which led to a slowed heart rate. Early management of DKA resulted in notable improvements in biological and clinical parameters, with hs-troponin levels decreasing.

**Figure 4:**
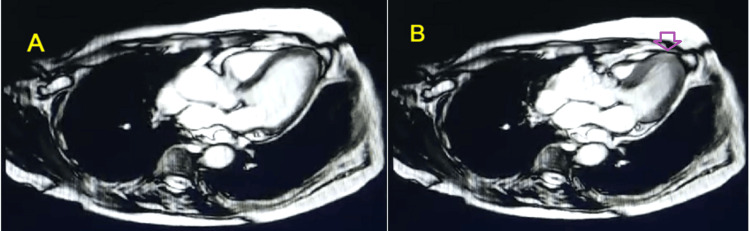
Axial MRI images in diastole (A) and systole (B) showing complete recovery of left ventricular function, with the arrow pointing to a normokinetic left ventricular apex

**Figure 5:**
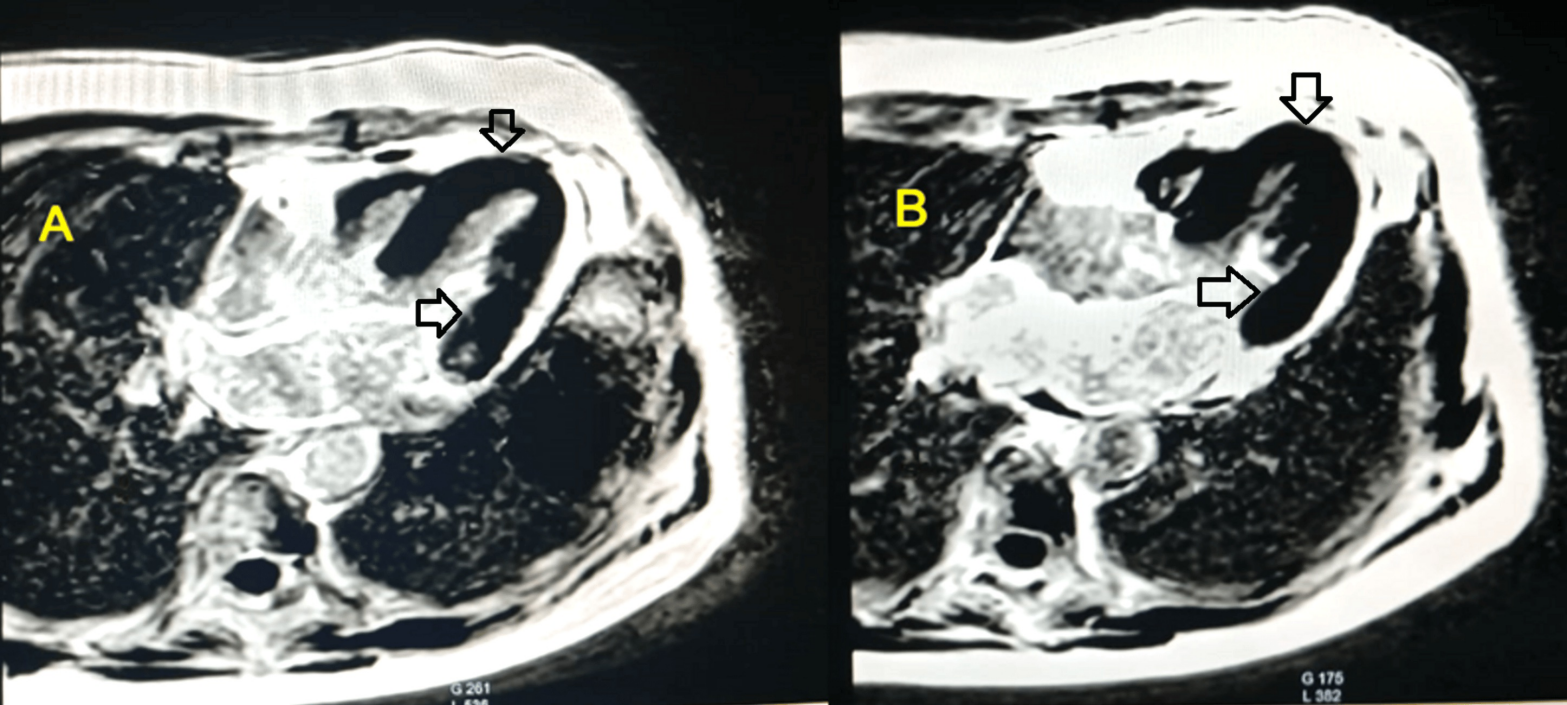
Axial MRI images showing the absence of late enhancement in the myocardial wall after gadolinium injection, in diastole (A) and systole (B), with arrows pointing to the left ventricular myocardial wall

A cardiac MRI scan performed one month after the event showed complete recovery of left ventricular function, without signs of myocarditis or ischemic damage, and no late enhancement. The ejection fraction was 62% (Figures 4A, 4B, 5A, 5B).

In addition, follow-up echocardiography performed several weeks later confirmed the resolution of left ventricular motion abnormalities. An EKG done two months after the event showed a sinus rhythm of 98 beats per minute with no repolarization abnormalities. A 24-hour Holter monitor conducted during the same period revealed a consistent sinus rhythm.

## Discussion

TTS, also known as stress cardiomyopathy, is increasingly recognized as a cause of acute chest pain and reversible left ventricular dysfunction, particularly in the absence of obstructive coronary artery disease [1,2]. Stress, whether physical or emotional, precedes the onset of symptoms in approximately 85% of cases. While the precise pathophysiology remains incompletely understood, several theories have been proposed, with catecholamine-induced myocardial stunning being one of the most prominent. Stress-induced catecholamine release is suggested to have a toxic effect on cardiomyocytes [3], and other contributing factors may include microvascular dysfunction, transient vasospasm, and metabolic abnormalities [3,4].

Initially considered a rare condition, TTS has gained widespread recognition and is now diagnosed globally. It predominantly affects postmenopausal women, although cases in men and younger individuals have been reported. Diagnosing TTS is challenging due to its clinical resemblance to acute coronary syndrome. Patients typically present with chest pain, dyspnea, and electrocardiographic changes that mimic ST-segment elevation myocardial infarction. However, the absence of significant coronary artery obstruction and the presence of transient left ventricular dysfunction are distinguishing features. Diagnostic tools such as echocardiography, cardiac MRI, and coronary angiography are essential for confirming the diagnosis and assessing cardiac function [5,6].

TTS and AF

AF has been linked to a higher risk of developing TTS, indicating a potential bidirectional relationship. Research by Hübscher et al. developed an in vitro model of ventricular cardiomyocytes with takotsubo, demonstrating their increased sensitivity to catecholamines and a decrease in catecholamine desensitization. Additionally, the team created in vitro models of atrial cardiomyocytes affected by TTS. They observed significant calcium homeostasis disorders and frequent atrial arrhythmias under adrenergic stress induced by isoprenaline, linked to decreased PDE4 activity [7,8].

Genotyping revealed that all patients had a mutation in the AHNAK gene, which regulates cardiac calcium channels. These patients exhibited higher intracellular calcium levels and lower AHNAK protein expression compared to controls without TTS. Using targeted gene therapies to create isogenic lines, the authors demonstrated that the AHNAK gene mutation leads to calcium hypersignaling and an increase in cells with irregular heart rates, associated with decreased PDE4 activity [7,8]. Another theory is that AF can elevate the heart’s workload, leading to rapid ventricular rates that increase myocardial oxygen demand and stress [8,9]. This heightened stress can trigger TTS, especially in patients with other risk factors, suggesting that AF could be a key precipitating factor in TTS onset [8,9].

TTS and diabetes mellitus

The relationship between diabetes mellitus and TTS is complex. Despite diabetes mellitus being associated with increased cardiovascular risks, paradoxically, diabetic patients may have a lower incidence of TTS compared to nondiabetics. A study by Thangjui et al., involving 155,000 patients undergoing cardioversion for AF, found a surprisingly lower prevalence of diabetes in TTS patients compared to those without TTS [10]. One hypothesis is that altered autonomic function in diabetic patients, including reduced sympathetic responsiveness and impaired catecholamine release, might attenuate the catecholamine surge that triggers TTS [10-14]. Furthermore, chronic hyperglycemia induces structural myocardial changes through advanced glycation end products and oxidative stress, potentially modifying the heart’s response to acute stress and mitigating myocardial stunning [12-14].

However, the association between DKA as an acute physical stressor and TTS remains to be thoroughly investigated. Walid et al. reported a case of recurrent TTS in a 56-year-old female triggered by repeated episodes of DKA [14]. This suggests that the physical stress associated with DKA, characterized by catecholaminergic discharge, may have a dominant effect, potentially counteracting the protective mechanisms of diabetes mellitus [14].

In our case, the patient’s postoperative course, complicated by DKA, AF tachycardia, and characteristic echocardiographic findings of apical akinesia, led to an early suspicion of TTS. The absence of significant coronary artery disease on angiography, further investigation with cardiac MRI, and complete recovery documented one-month post-event confirmed the diagnosis of takotsubo cardiomyopathy. This case highlights the intricate association between TTS, diabetes mellitus, and AF tachycardia. Understanding the multifaceted interactions between takotsubo cardiomyopathy, AF, and diabetes mellitus remains crucial. The bidirectional relationship between TTS and AF presents new avenues for research and clinical management.

## Conclusions

We report a case of TTS in a 76-year-old female patient who experienced a persistent episode of DKA and AF tachycardia, which spontaneously resolved. This case highlights the complex and intriguing relationship between TTS, diabetes mellitus, and AF. Although diabetes mellitus is generally considered a protective factor against TTS, episodes of DKA may act as triggers. AF is more likely to occur during TTS due to decreased PDE4 activity, altered calcium homeostasis, and increased sensitivity to catecholamines. In our case, AF resolved spontaneously one month after the event. Further research is needed to investigate the reduction of AF following TTS and to clarify the mechanisms linking TTS with diabetes mellitus and AF.
